# The stiffness adjustable wheel mechanism based on compliant spoke deformation

**DOI:** 10.1038/s41598-024-51493-x

**Published:** 2024-01-08

**Authors:** Hyeungyu Yoon, KangYub Lee, Jongmyeong Lee, Joonhyuk Kwon, TaeWon Seo

**Affiliations:** School of Mechanical Engineering, Hanyang University, Seoul, 04763 Republic of Korea

**Keywords:** Engineering, Mechanical engineering

## Abstract

This study proposes a variable-stiffness mechanism for non-pneumatic tires such that can actively adapt to various environments. Non-pneumatic tire is a compliant wheel structure that offers superior robustness and adaptability compared to pneumatic tires. However, the tire designed for certain terrain exhibits relatively high rolling resistance and inadequate suspension. To address these problems, a stiffness-adjustable wheel (SAW) that can modify the force applied to the contact surface is introduced in this study. In addition, the shape of SAW is optimized to maintain a desirable range of stiffness under different conditions. The optimization is conducted with experimental method, because nonlinear response of material and interference between components make it difficult to predict the characteristic of the wheel at large deformation. The SAW has potential for application in various mobile platforms to provide adequate stiffness for a variety of terrains and driving conditions.

## Introduction

A tire is a part of the wheel that absorbs vibrations and impacts, and a pneumatic tire, which uses air, is the most common type. A tire endows the wheel the capacity to buffer against impacts and ensures smooth responses. Unlike rigid wheels, pneumatic tires minimize energy loss on rough surfaces and feature low contact pressure, low perpendicular stiffness, and low mass^1,2^. However, pneumatic tires also have disadvantages such as vulnerability to deflation and the need for pressure maintenance.

Non-pneumatic tires (NPTs) were developed as an alternative to their pneumatic predecessors, replacing air with elastic material spokes and a rim to form a spring wheel or airless tire^3–5^. Each spoke resists wheel deformation, unlike air pressure in pneumatic tires, as the stress on the spokes is more localized. This also enables an NPT to modify the driving characteristics^6^.

An NPT does not require air pressure management, making it robust against damage and leakage^1,7,8^. So NPT is used for mobile robot that operate in harsh environment. For example, pneumatic tires experience significant pressure changes during space travel, and the operation of vehicles such as a planetary exploration rover cannot be restored on another planet if the wheels have punctures. Therefore, several rovers have adopted NPTs, and extensive research has been conducted on their design^9–11^. NPTs are also chosen for their longer service life. Michelin’s product, Tweel, has three times the service life, and greater efficiency, than conventional pneumatic tires. Michelin also claims that Tweel can reduce fuel consumption and waste, making it an eco-friendly option^12,13^.

NPT wheels also can be designed to drive over obstacles by suppressing impact and vibration, especially they are made of soft materials^7,8^. The shape of the spokes determines the features of the wheel, allowing engineers to select the appropriate reaction of the wheel when encountering obstacles. The spokes deform to adapt to the surface, thereby distributing the force and enabling the mechanical elastic wheel to achieve a smooth response on different terrains. NPT wheels, including mechanical elastic wheels, are mechanisms with potential applicability in various fields^14^.

However, NPT wheels have a critical disadvantage. Their design, which is optimized for a smooth response, results in low perpendicular stiffness. Although these characteristics are beneficial for overcoming obstacles and reducing the risk of wheel pitting (e.g., when driving in sand) because the load is distributed on the ground, they reduce driving efficiency on well-paved flat surfaces such as regular roads^15^ because of rolling resistance. Moreover, soft wheels inherently have a large deformation range, and their contact force perpendicular to the contact surface has a nonlinear relation with the deformed length, owing to interference from structural components^16,17^. Various studies have attempted to optimize the wheel shape to achieve both high stiffness and response smoothness while maintaining a linear spring constant^2,18–20^. However, these studies did not address differing terrain conditions. In addition, when the payload changed, the wheel’s characteristics deviated from the design purpose.

Stiffness is the extent to which an object resists deformation in response to an applied force. For wheels, stiffness is equal to the contact force divided by the deformation and the relationship between stiffness and contact force can be considered divided according to the tangential and perpendicular directions. Perpendicular stiffness directly affects the rolling resistance in wheels, with higher stiffness resulting in lower resistance. There are researches show that the rolling resistance is related with stiffness^21^ and the energy consumption is induced from deformation cycle^22–24^ on flat and rigid surface. However, at sand, the rigid wheel make larger rolling resistance digging soil because of concentration of pressure^25,26^. In case of rough terrain, to obtain a smooth response, a large deformation range is required^27,28^ and it has been studied that the stiffness can vary in a small deformation range depending on the roughness of the surface^29^. This limits the extent by which the stiffness of the wheels can be increased. As a result,the conventional wheels and tires are optimized for certain terrain but fixed-shape wheels cannot effectively respond to various forces generated by velocity, payload, and impact caused by falling and landing. Therefore, mechanisms that can actively respond to various situations should be explored by balancing these two factors in one wheel.

**Figure 1:**
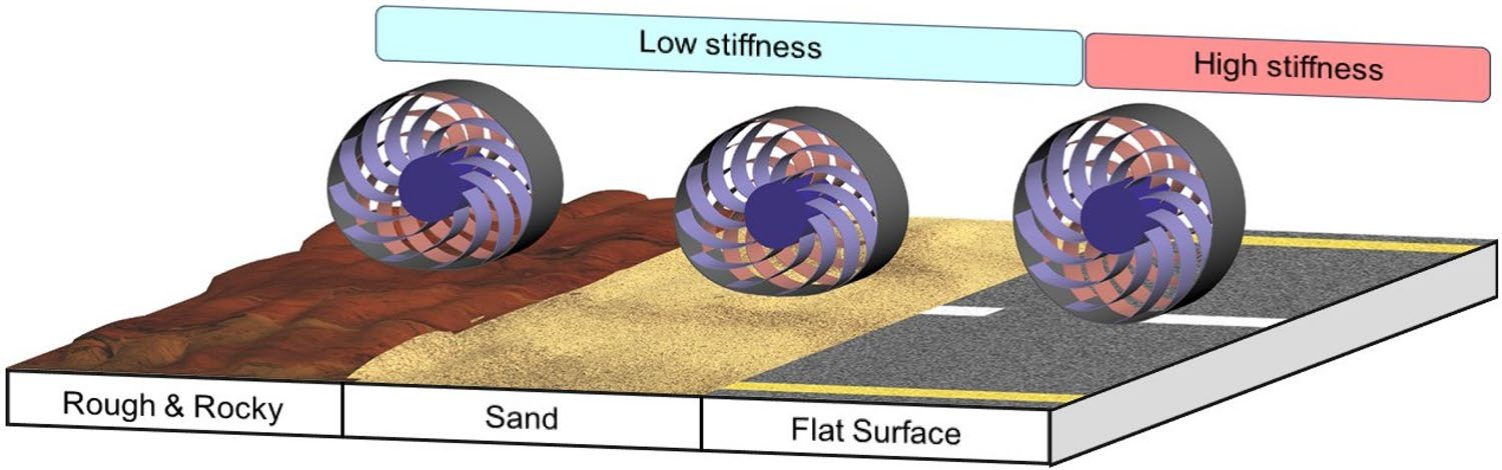
The concept of the SAW coping with various terrains, on (1) rough and rocky, (2) sand (3) and flat surface.

**Figure 2:**
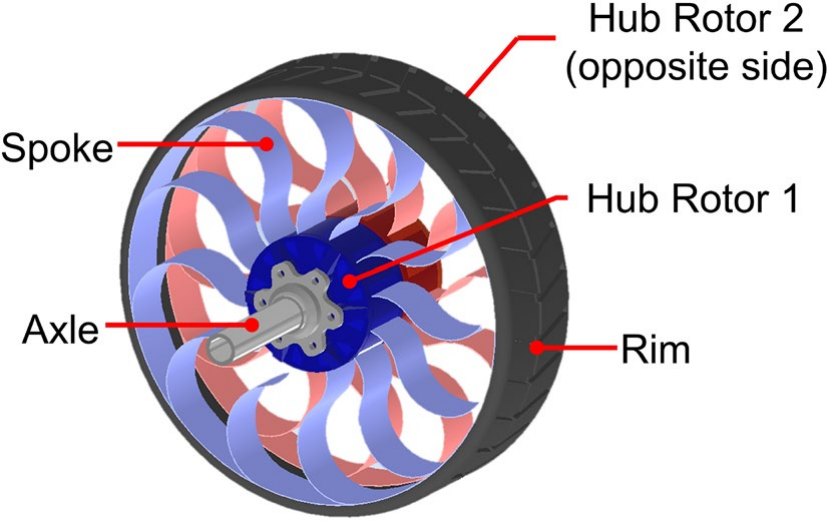
Design and configuration of the proposed SAW.

This study aims to improve the response smoothness of NPT wheels by addressing three limitations: (1) high rolling resistance caused by low perpendicular stiffness, (2) nonlinear spring constant, and (3) inadequate responsiveness to various forces. Hence, this study introduces a stiffness-adjustable wheel (SAW) as a novel NPT mechanism that actively varies the perpendicular contact force based on the surface and operating conditions. As shown at Fig. 1, (1) the SAW decrease its perpendicular stiffness at rough and rocky terrain to absorb shock. (2) And also decreased perpendicular stiffness is helpful on sand, because it make wheel to have larger contact area preventing digging the sand. (3) On flat surface, the SAW increase its perpendicular stiffness to reduce rolling resistance induced by deformation. Figure 2 illustrates the overall structure of the proposed SAW. As the NPT characteristics are determined by the structural shape and stress, the SAW can alter its characteristics by changing the structural shape during operation. For example, when driving on a flat surface at high speeds, perpendicular stiffness of the SAW increases to reduce the rolling resistance. On the contrary, when encountering a rough terrain, the stiffness of the SAW decreases to ensure a smooth response and to reduce vibrations and impacts. Overall, the SAW can behave as an active suspension system by adapting to various terrains, forces, and speeds.

This study introduces new compliant wheel mechanism, the SAW. The SAW mechanism can change perpendicular stiffness to make mobile robot to cope with the various terrain. Therefore maintaining the ability to change the stiffness under a wide range of terrain shape and deformation depth is necessary. In this study, the deformation range was set up to one third of the wheel radius, to be half the thickness of the tire which is larger than the deformation amount of general NPT^8,13^. The design and underlying mechanisms of SAW is explained in next section. Experimentation is essential for research, as it unveils the interference between substructures and exposes the statically indeterminate problems. Based on the SAW design concept, the object function and experimental method are defined, along with an explanation of the test environment. As a result, design parameter perpendicular stiffness measured at optimized model will be presented. Finally, the conclusions drawn from the study findings are presented.

## Stiffnes adjustable wheel (SAW) mechanism with one degree of freedom

This section describes the configuration of SAW and how the mechanism works.

### Concept of the mechanism

**Figure 3:**
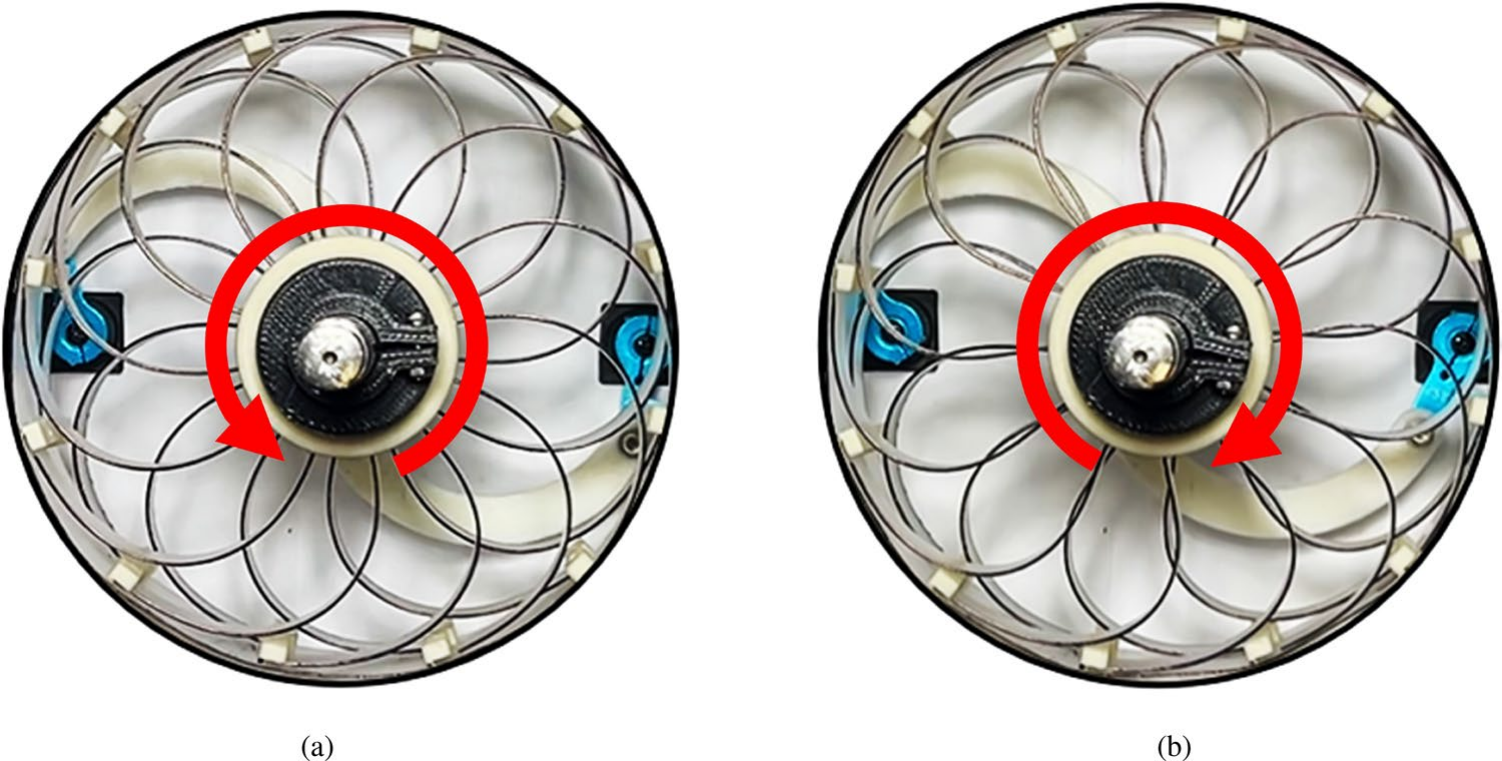
Rotation of rotor and the spoke shape of SAW. (**a**) Counterclockwise rotor motion, decreasing perpendicular stiffness. (**b**) Clockwise rotor motion, increasing perpendicular stiffness.

The SAW uses two rotors on the driving axis to modify the shape of the spokes and adjust their perpendicular stiffness. The spokes are connected to a central rotor. One side of this rotor is fixed to the wheel axle, and the other side has the freedom to drive against it. Rotating rotors of both side to opposite direction for each other, it make spoke move and deformed, while the rim constrains the tips of each spoke. As shown in Fig. 3, when the spokes are deformed, the relative position between their tips and the overall radius of the wheel remains unchanged. Consequently, only the curvature of spoke is varied to modify the stiffness of the SAW. The spokes are constructed using sheet material to ensure a uniform stiffness variation throughout the structure. This type of plates is preferred to complex shaped plates for exploration rovers or military robots owing to its reliability and consistent physical properties. Additionally, it allows the use of metals with stable changes in physical properties according to the intended environment^30^. The deformation-perpendicular contact force force curve of a conventional NPT is the way often used to present the wheel characteristics. On the other side, the SAW can change the perpendicular contact force force at specific deformations, it exhibits a deformation-perpendicular contact force force surface instead of the curve, which represents its unique characteristics.

### Relationship between spoke curvature and force

**Figure 4:**
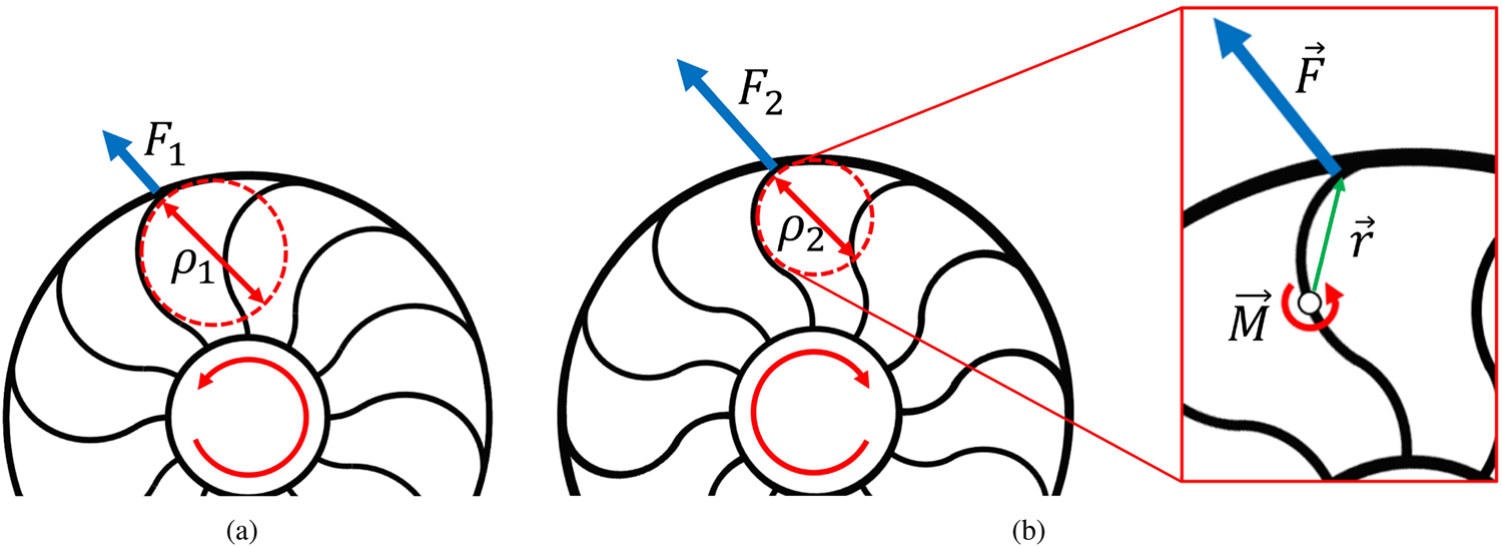
Schematic of the relationship between spoke curvature and perpendicular stiffness for the SAW mechanism. (**a**) Curvature for decreased the stiffness. (**b**) Curvature for increased the stiffness and magnified view of a spoke.

The position of the spoke tip determines the spoke curvature, which generates a force at the tip. In the SAW, the rotor alters the position of the spoke tip, which in turn changes the stress distribution within the structure. As depicted in Fig. 4, the motion of the rotor deforms the spokes and changes the curvature of spoke from $\rho _1$ to $\rho _2$. Curvature of spoke $\rho$ is related to moment *M*, Young’s modulus *E*, and area moment of inertia *I*. Let the $\vec {r}$ is the vector from a point on spoke to rim. The force at the spoke tip is affected by the moment and $\vec {r}$. Consequently, the rotor motion changes the curvature and moment on spoke, enabling the SAW to adjust its perpendicular stiffness.

There are several difficulty at predicting characteristics of the SAW with analytic method, because of (1) the analysis of fixed-tip spokes involving statically indeterminate problems including various mode at shape, (2) the interference between the sub-structures, (3) nonlinear strain–stress response of most materials at large deformation. The numerical analysis also (4) requires initial shape and constraint force for initial and boundary conditions, which only can be obtained from analytic prediction^31^. Because of these four reasons, the experiment was inevitable to figure out the characteristics of the SAW. As experimental shape optimization requires to fabricate the wheel samples, it was performed using the Taguchi method, which is efficient given the reduced number of required test cases^32^.

## Experimental setup

### Object function and user conditions

**Figure 5:**
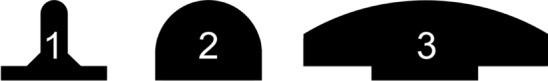
Considered terrain shapes with circular shapes of 10 mm (1), 40 mm (2), and 160 mm (3) in radius.

The SAW was developed to address various terrain conditions, velocities, and payloads. The velocity and payload influence the range of deformation, whereas the terrain affects the shape. In this study, the user conditions were defined as the displacement of the contact surface caused by deformation and the shape of the surface. As permanent deformation of the prototype started at 30 mm, the deformation was set to 5 mm, 15 mm, and 25 mm to test SAW perpendicular stiffness while avoiding damage. To mimic various terrain shapes, circles with radius of 10 mm, 40 mm, and 160 mm were considered as depicted in Fig. 5, and the user conditions are summarized at Table 1. Under these deformation and terrain conditions, the SAW was required to exhibit a wide range of perpendicular contact force forces or perpendicular stiffness to control the host platform acceleration. The object function ($f_{obj}$) can be defined as Eq. (1), which represents the normalized range of the force under the given user conditions by using measured maximum and minimum perpendicular contact force force($F_{max}, F_{min}$). Because the range of required the stiffness may vary depending on the wheel’s application, which can be amplified with the same characteristics by changing the material of the wheel or proportionally adjusting the thickness and width of the spokes and rim.

1 $$\begin{aligned} f_{obj} = \frac{{F_{max}}-{F_{min}}}{F_{max}} \end{aligned}$$

**Table 1:** User conditions for experimental optimization in three levels.

User conditions	Level		
	1	2	3
Radius of obstacle	10	40	160
Deformation	5	15	25

### Test bench design

**Figure 6:**
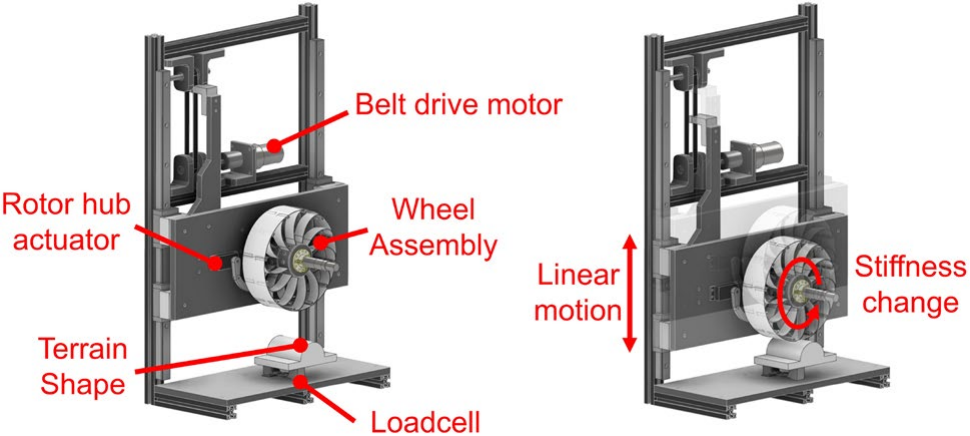
Configuration (left) and operation (right) of test bench.

A test bench was developed to measure the perpendicular contact force force of the wheels. The test bench consisted of a linear transfer motion mechanism that caused deformation length and a terrain block placed against a wall to affect the deformation shape. The force applied to wheel was measured using a load cell positioned between the terrain block and wall. A servo motor located below the wheel operated each rotor in opposite directions, thereby changing the wheel stiffness. The configuration and operation of the test bench are depicted in Fig. 6.

### Design parameters

Assuming that the wheel operates in a two-dimensional space excluding the rotation axis direction, the design factors that can affect perpendicular stiffness are *R* (wheel radius), $I_{s}$ (spoke area moment of inertia), $I_{r}$ (rim area moment of inertia), *L* (spoke length), *r* (rotor radius), $\alpha _{1}$ (angle between spoke and rotor), and $\alpha _{2}$ (angle between spoke and rim). Rotor radius *r* and wheel radius *R* is excluded as a design parameter, given the implementable size limitation. The wheel and rotor diameter set as 150 mm and 50 mm. The other factors are selected as design parameters. As the section of spoke is rectangular, the area moment of inertia can be adjusted by changing the thickness and width of spokes, as shown at Eq. (2). The *t* is thickness and *w* is width of spokes.

2 $$\begin{aligned} I_{rectangular} = \frac{wt^3}{12} \end{aligned}$$

**Figure 7:**
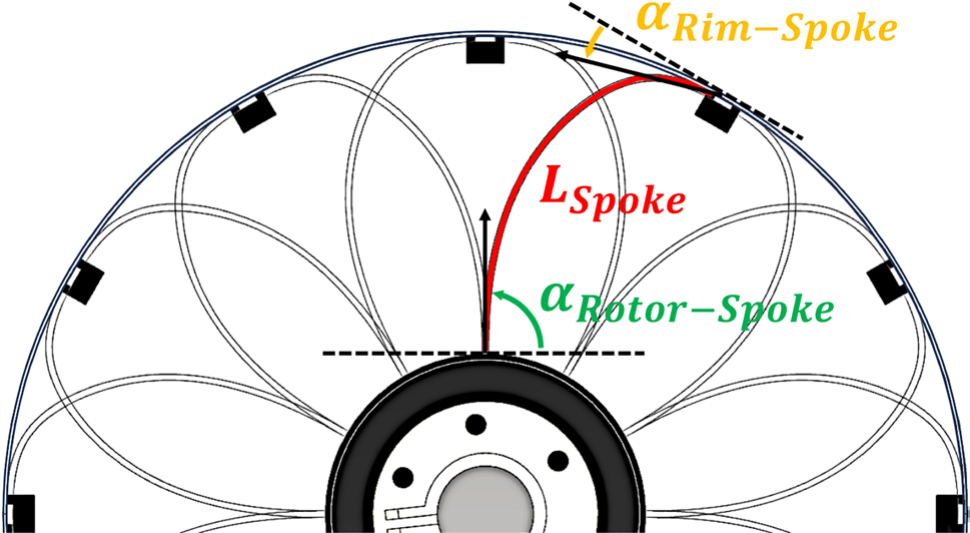
Diagram of SAW design parameters.

The characteristics of wheel affected by the area moments of inertia have static similarity. Thus, the design parameters can be reduced by setting ratio $\frac{I_{r}}{I_{s}}$. The resulting model may allow control over the overall SAW stiffness by changing moments of inertia (*I*) of the spoke and rim while maintaining their ratio. Figure 7 depicts the design parameters of the SAW, and Table 2 lists the possible parameter values in three levels. The parameter values are set as wide as possible in the range that the wheel can operate and maintain its shape. Since there is no mathematical model, they had to be found empirically. If the spoke length was too short, it was difficult to connect from the rotor to the rim, and if it was too long, they overlapped on the rim, creating a stiffness dead zone. So, when we first created the prototype, we overcame these problems and found a workable length (approximately 80 mm), and based on this, we set intervals of 10 mm as the initial range. In case of angles, there were relatively few restrictions on them, the range of value was set to run as many cases as possible. As the shape of the SAW is 2-dimensional problem, if the spokes are attached at 30$^{\circ }$ to the rim and 60$^{\circ }$ to the rotor, this is the same as 150$^{\circ }$ to the rim and − 60$^{\circ }$ to the rotor. So, the range was set from 0$^{\circ }$ to 90$^{\circ }$ from the tangential direction relative to the rim, and from − 45$^{\circ }$ to 45$^{\circ }$ to the rotor. The reason why rim-spoke angle was not set as from − 90$^{\circ }$ to 90$^{\circ }$ is that the spokes make too much interference at the angles.

**Table 2:** Design parameter values in three levels.

Design parameter	Level		
	1	2	3
Rim-spoke angle (deg)	0	30	60
Rotor-spoke angle (deg)	− 45	0	45
Spoke length (mm)	70	80	90
I ratio	0.3	0.6	0.9

## Optimization by Taguchi method

Conducting experiments for all the combinations of design parameters would be inefficient and time-consuming, as 81 ($3^4$) cases of the wheel would need to be considered, with each case requiring 9 ($3\times 3$) measurements for all the usage conditions. Instead, the Taguchi method can be applied to reduce the number of required experiments. This method is intended to make products or processes robust under various conditions while maintaining their performance. By using orthogonal arrays, the Taguchi method allows to reduce the number of experiments while enabling a sensitivity analysis of each design parameter^32^.

The signal to noise(S/N) ratio is key factor at Taguchi method. Signal refers to the response characteristics of the mechanism we expect, and noise refers to variables that vary depending on the environment. Therefore, a high S/N ratio means that the signal response characteristics are greater than those of noise, which means that it has robust characteristics against various environmental variables. The S/N ratio was calculated using Eq. (3). The $f_{obj}{(n,m)}$ is object function value calculated from given user condition. The *n* is the number of user condition for terrain shape and the *m* is for deformation. Since they each have 3 levels, a total of 9 ($3\times 3$) object functions are calculated for the S/N ratio.

3 $$\begin{aligned} S/N\,ratio = -10\log |\frac{{\left( {\frac{1}{f_{obj}{(1,1)}}}\right) ^2}+{\left( {\frac{1}{f_{obj}{(1,2)}}}\right) ^2} +\cdot \cdot \cdot +{\left( {\frac{1}{f_{obj}{(n,m)}}}\right) ^2}}{n\times m}|\;[dB] \end{aligned}$$

### Experimental procedure

**Figure 8:**
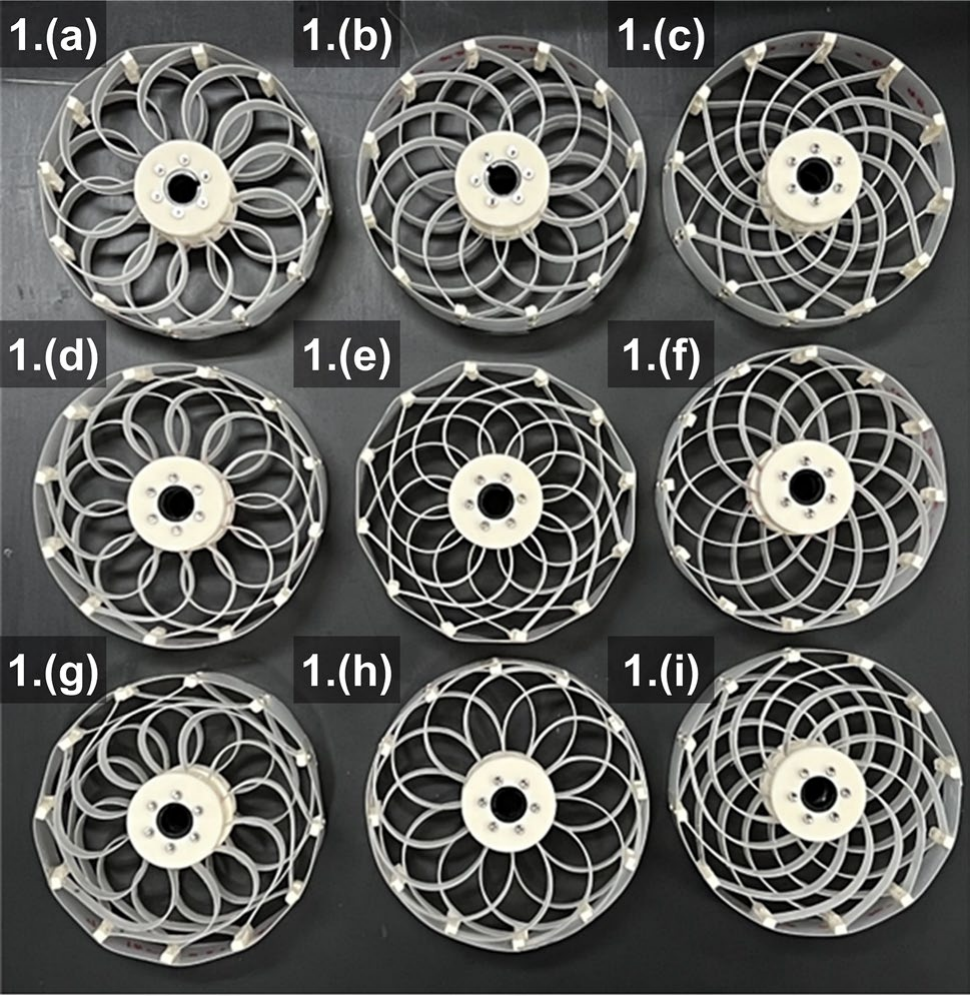
Nine wheel samples fabricated for first experiment.

The orthogonal array of design parameters for experimentation is presented in Table 3. Each combination is tested under the nine conditions. Figure 8 shows the SAW samples obtained from the combinations listed in Table 3.

**Table 3:** Orthogonal array of design parameters for the first experiment.

Test 1	Design parameter			
	Spoke length (mm)	Rotor-spoke angle (deg)	Rim-spoke angle (deg)	I ratio
a	70	− 45	0	0.3
b	70	0	30	0.6
c	70	45	60	0.9
d	80	− 45	30	0.9
e	80	0	60	0.3
f	80	45	0	0.6
g	90	− 45	60	0.6
h	90	0	0	0.9
i	90	45	30	0.3

**Figure 9:**
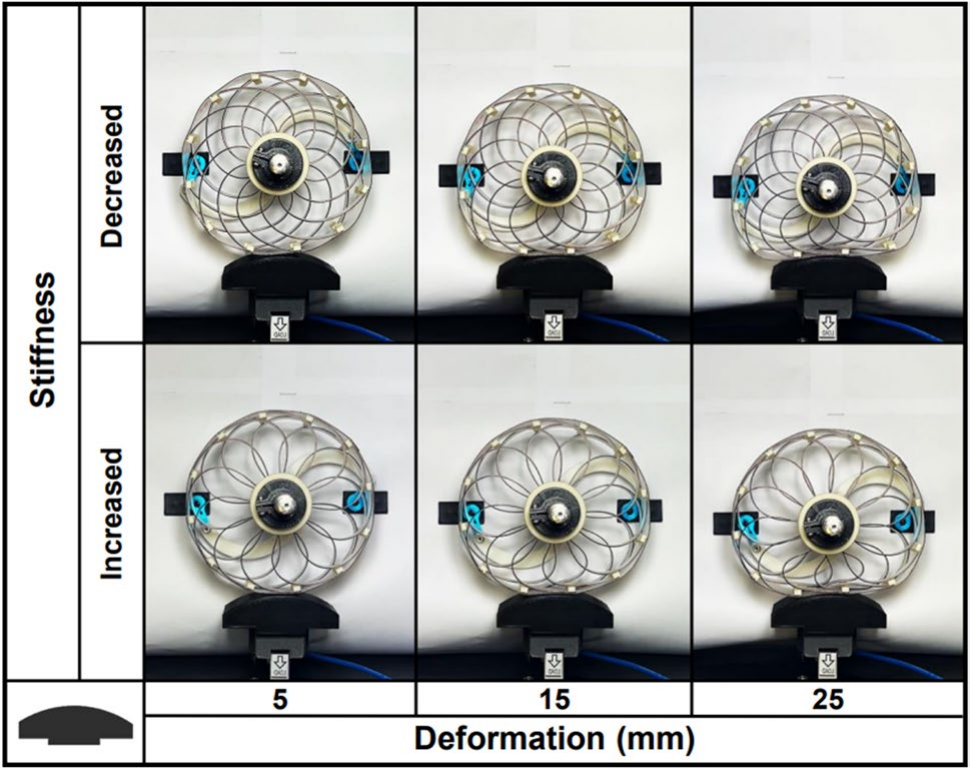
Experiment for SAW sample 1.(f) on terrain shape 3.

Each SAW sample was tested in accordance with the described procedure under nine conditions. On the test bench, the wheel is pressed at three deformation depths for each of the three terrain shapes, and the perpendicular contact force is measured by load cell while rotating the rotor at the center of the wheel. As the rotor rotates, the shape of the spokes changes resulting in a change in vertical contact force, and the maximum and minimum forces were then recorded. The range of rotor motion was limited by the angle at which the wheel lost its self-centering ability. Figure 9 illustrates the experimental procedure and wheel shape for one terrain shape. The results and S/N ratio are listed in Table 4.

**Table 4:** Measured forces and S/N ratio in first experiment.

Test 1		Load cell data (kgf)						S/N ratio (dB)
	Terrain shape	1		2		3		
	Deformation (mm)	max	min	max	min	max	min	
a	5	0.49	0.36	0.38	0.25	0.43	0.37	− 14.19
	15	2.50	1.77	1.74	1.25	2.11	1.73	
	25	4.61	3.95	4.31	3.49	4.81	3.94	
b	5	0.24	0.21	0.23	0.21	0.22	0.11	− 19.29
	15	0.65	0.61	0.65	0.61	0.63	0.47	
	25	1.72	1.02	1.83	1.00	1.82	1.25	
c	5	0.21	0.20	0.21	0.20	0.18	0.18	− 35.77
	15	0.50	0.45	0.60	0.41	0.43	0.40	
	25	1.07	0.82	1.30	0.88	1.12	0.76	
d	5	0.16	0.14	0.14	0.14	0.13	0.13	− 36.15
	15	0.73	0.33	0.67	0.30	0.67	0.34	
	25	1.49	1.07	1.49	0.92	1.40	0.78	
e	5	0.39	0.32	0.44	0.36	0.39	0.32	− 16.92
	15	1.29	0.85	1.41	1.00	1.25	1.18	
	25	3.58	1.55	3.52	1.64	3.41	1.95	
f	5	0.46	0.14	0.36	0.16	0.58	0.17	− 5.645
	15	1.41	0.60	1.20	0.44	1.43	0.64	
	25	2.28	1.29	2.20	1.25	2.20	1.32	
g	5	0.29	0.13	0.20	0.10	0.19	0.12	− 13.18
	15	1.16	0.75	1.05	0.44	0.96	0.35	
	25	2.12	1.90	1.93	1.69	2.01	1.21	
h	5	0.24	0.19	0.26	0.20	0.33	0.09	− 12.81
	15	0.76	0.62	0.79	0.65	0.93	0.41	
	25	1.64	1.33	1.55	1.18	1.67	1.28	
i	5	0.40	0.18	0.32	0.20	0.33	0.29	− 10.64
	15	1.55	0.69	1.41	0.79	1.54	1.01	
	25	3.48	1.41	3.08	1.33	3.27	1.44	

**Figure 10:**
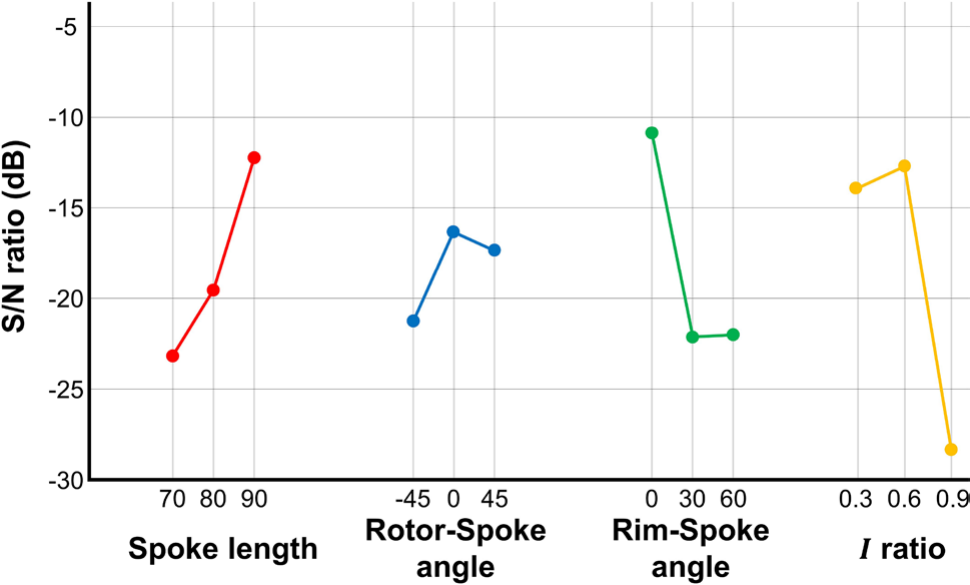
Average S/N ratio according to different parameters for first experiment.

By comparing the average S/N ratio per factor, the effects of the design parameters can be analyzed. Figure 10 shows the parameter effects on the SAW performance. The angle between the rim and spoke ($\alpha _{2}$) reaches the highest S/N ratio at 0$^\circ$. This value is considered as the optimal one because values below 0 indicate that the spokes go out of the wheel, resulting in an unfeasible shape. The angle between the rotor and spoke ($\alpha _{1}$) converges and shows low sensitivity, with the value of 0 $^\circ$ considered as optimal. On the other hand, the spoke length (*L*) shows improvement as it increases. As it exhibits high sensitivity and does not converge, additional experiments are required for a longer range.

### Additional second experiment

**Figure 11:**
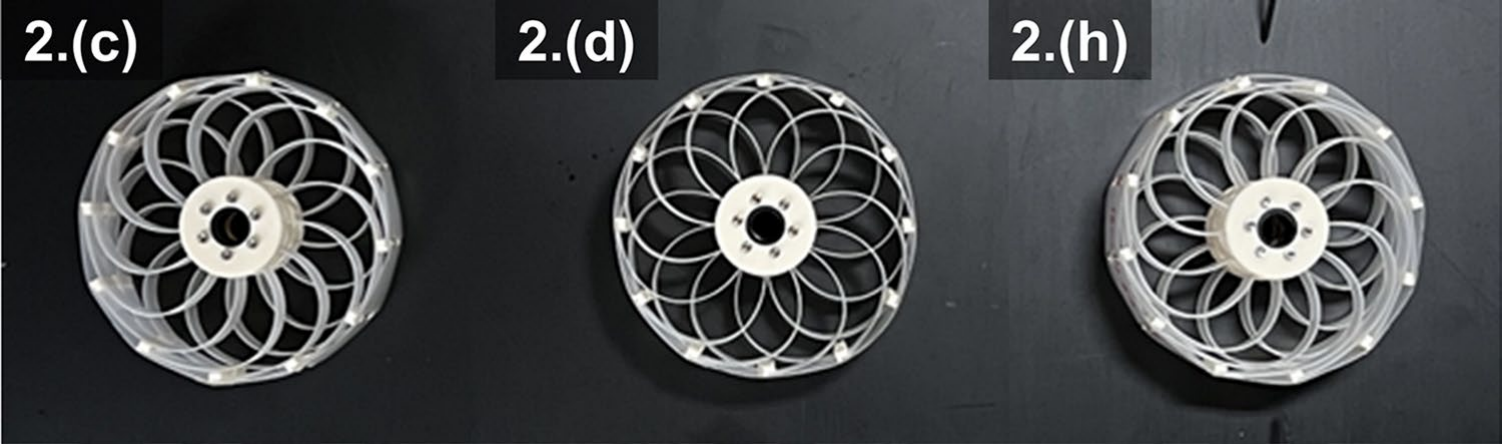
Additional samples fabricated for second experiment.

For the second experiment, the spoke lengths were set to 90 mm, 105 mm, and 120 mm. The ratio of the area moment of inertia ($\frac{I_{r}}{I_{s}}$) converges, but it shows high sensitivity, and the difference between 0.6 and 0.9 is too significant to conclude convergence. Therefore, an additional parameter of 0.75, which is the median of those two values, is evaluated. The rotor-spoke and rim-spoke angles were fixed to 0$^\circ$. The second experiment involved two design parameters with three levels per parameter, resulting in nine combinations. Table 5 lists these combinations, and Fig. 11 shows the three representative SAW samples for the second experiment.

**Table 5:** Design parameter values for second experiment.

Test 2	Design parameter	
	Spoke length (mm)	I ratio
a	90	0.3
b	90	0.6
c	90	0.75
d	105	0.75
e	105	0.3
f	105	0.6
g	120	0.6
h	120	0.75
i	120	0.3

**Figure 12:**
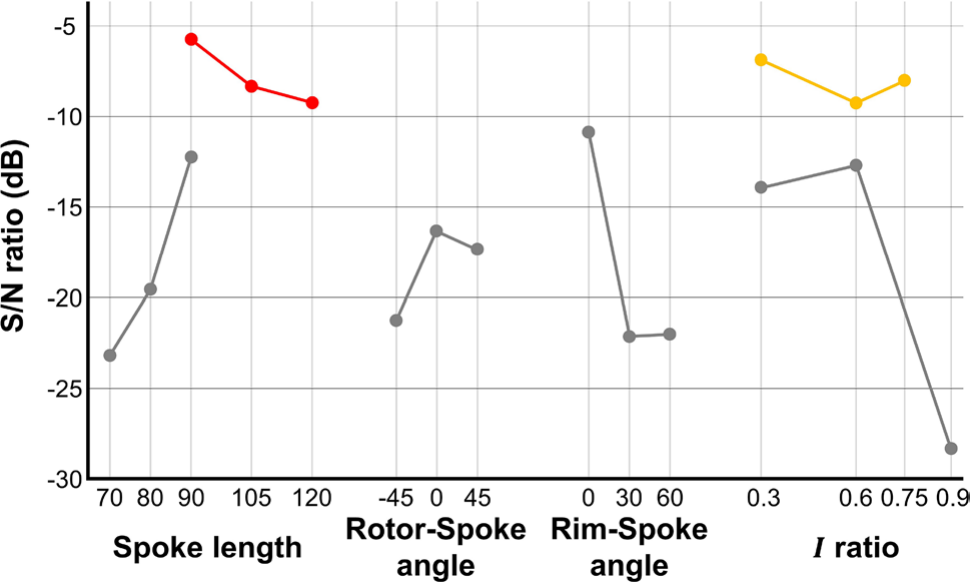
Average S/N ratio according different parameters for the second experiment and results from the first experiment for comparison.

The second experiment followed the same procedure as the first one, and its results are listed in Table 6. Comparing the S/N ratio according to each parameter with the results of the first experiment, an improvement can be observed. Figure 12 shows the S/N ratio according to the parameters for the second experiment along with that for the first experiment shown as gray dots and lines. The spoke length shows a decreasing S/N ratio trend after 90 mm. As lengths shorter than 90 mm exhibit lower S/N ratios in the first experiment, 90 mm can be considered as the optimal spoke length. The area moment of inertia ratio exhibits a different curve compared with the first experiment, showing the *I* ratio 0.6 has the lowest S/N ratio and 0.3 has the highest one while the optimal value is 0.75. This phenomenon is known to occur when this design parameter does not have an independent effect on other design parameters. The change in trend is seemed to be influenced by the spoke shape. But the sensitivity is smaller than the other design parameters, indicating a negligible effect, it was decided to complete the experimental optimization process.

**Table 6:** Measured forces and S/N ratio in the second experiment.

Test 2	Terrain shape	Load cell data (kgf)						S/N ratio (dB)
		1		2		3		
	Deformation (mm)	max	min	max	min	max	min	
a	5	1.05	0.24	0.99	0.22	0.85	0.22	− 4.784
	15	2.65	1.15	2.75	1.15	2.67	0.86	
	25	4.76	2.50	4.41	2.23	4.13	2.31	
b	5	0.49	0.19	0.37	0.14	0.43	0.12	− 7.953
	15	1.37	0.86	1.34	0.82	1.20	0.47	
	25	2.47	1.86	2.42	1.58	2.06	1.32	
c	5	0.50	0.11	0.50	0.10	0.41	0.09	− 4.678
	15	1.21	0.62	1.18	0.57	1.15	0.48	
	25	2.44	1.17	2.42	1.20	2.03	0.87	
d	5	0.24	0.10	0.22	0.10	0.18	0.08	− 8.695
	15	1.06	0.63	1.04	0.61	0.97	0.56	
	25	2.20	1.68	2.20	1.61	2.26	1.45	
e	5	0.56	0.22	0.38	0.14	0.43	0.19	− 6.332
	15	2.41	1.24	2.11	0.91	1.93	0.83	
	25	4.25	2.91	4.21	2.43	3.97	2.03	
f	5	0.25	0.17	0.24	0.18	0.25	0.13	− 9.858
	15	1.26	0.65	1.24	0.72	0.92	0.73	
	25	2.77	1.93	2.81	1.72	2.37	1.41	
g	5	0.34	0.17	0.34	0.17	0.41	0.14	− 9.924
	15	1.34	0.81	1.45	0.81	1.31	0.67	
	25	2.39	1.780	2.53	1.96	2.17	1.47	
h	5	0.42	0.19	0.42	0.19	0.33	0.07	− 10.72
	15	1.33	1.00	1.44	0.91	1.27	0.63	
	25	2.39	1.98	2.40	1.87	2.12	1.59	
i	5	0.94	0.34	0.71	0.26	0.57	0.15	− 9.675
	15	2.91	2.01	2.71	0.56	2.55	0.42	
	25	5.31	4.46	4.83	3.49	4.36	3.06	

The second experiment, by investigating all possible combinations, was conclusive for the optimization experiment. Wheel design 2.(c) provided the highest S/N ratio (− 4.678 dB), indicating better performance under various conditions compared with the other designs.

## Result and discussion

### Result of optimization

**Figure 13:**
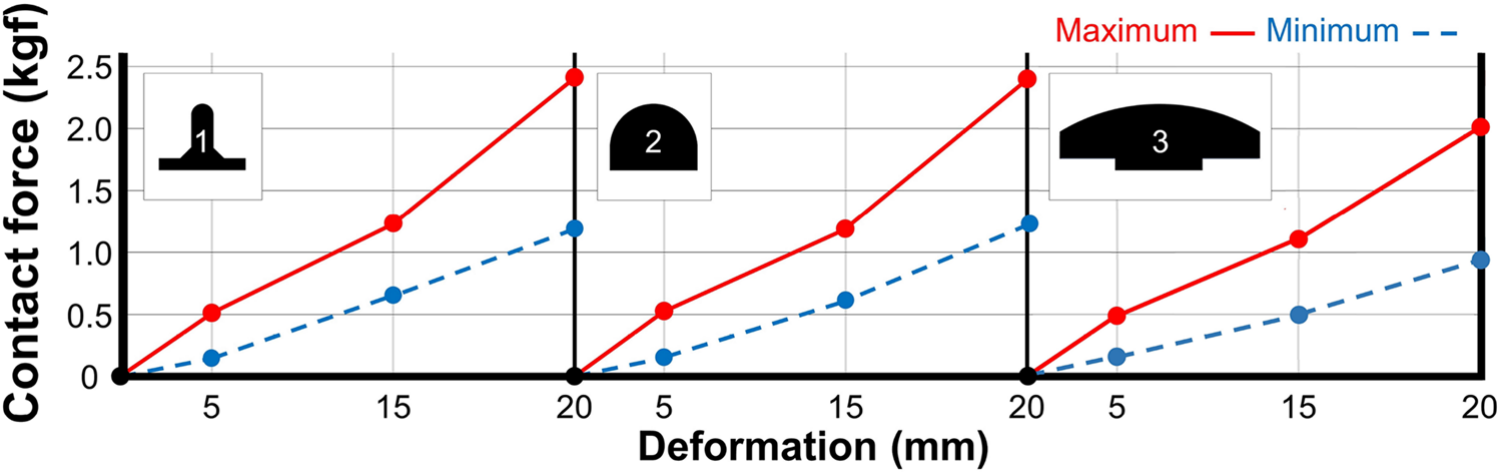
Measured perpendicular contact force force of wheel design 2.(c) according to terrain shape and deformation depth.

Through experimental optimization, wheel design 2.(c) from the second experiment was identified as the optimal one. The optimal design value is represented at Table 7. At future work, it can be enlarged or reduced while maintaining proportions for designated purpose, excepting I ratio because it is already proportional value. Figure 13 shows the perpendicular contact force according to the terrain shape and wheel deformation for the optimal wheel. Minimal performance degradation under different conditions and a wider range of perpendicular stiffness variation are achieved, compared with the other designs.

**Table 7:** Optimal design values for the SAW mechanism.

Design parameter	Spoke length	Rotor-spoke angle	Rim-spoke angle	I ratio
Optimized value	90 mm	0 (deg)	0 (deg)	0.75

## Conclusion

We introduce an NPT mechanism called the SAW, which can adjust its perpendicular stiffness during motion to adapt to different environmental conditions. The SAW can traverse over surfaces like flat terrains, sand, and rough terrains, which require different levels of deformation. The design concept of the SAW mechanism is explained, highlighting the factors that can influence its performance. Through experimental optimization, we identify the appropriate design parameters to maintain optimal performance under different conditions. The optimized model was fabricated and tested using the same experimental setup. The results obtained at maximum and minimum perpendicular stiffness values may guide future research.

However, the mathematical model that can explain and predict the characteristic of the SAW is not developed, so the following up study about the model is on going. And there is several characteristic must be improved. The visco-elasticity also can affect on the performance of wheel.

Another direction for future research is the active control of the SAW mechanism, which can be considered as an active suspension capable of controlling the kinetic energy of the body. The SAW can actively absorb impacts and vibrations. In addition, the passive characteristics of the SAW can also be explored. Increased rolling resistance led to a softer response when the rotor was not fixed. Therefore, passive mechanisms can be developed using the SAW concept.

### Supplementary Information

Supplementary Information 1.

## Data Availability

All data generated or analysed during this study are included in this published article and its Supplementary Information files.
